# 4,4′-Dimethyl-1,1′-(*p*-phenyl­enedimethyl­ene)dipyridinium bis­[7,7,8,8-tetra­cyano­quinodimethanide(1−)]

**DOI:** 10.1107/S1600536810017113

**Published:** 2010-05-15

**Authors:** Guang-Xiang Liu

**Affiliations:** a Anhui Key Laboratory of Functional Coordination Compounds, School of Chemistry and Chemical Engineering, Anqing Normal University, Anqing 246003, People’s Republic of China

## Abstract

In the title salt, C_20_H_22_N_2_^2+^·2C_12_H_4_N_4_^−^, the cations and anions stack along the *b* axis into segregated columns. In the cation, which has a crystallographically imposed centre of symmetry, the dihedral angle between the benzene and pyridine rings is 89.14 (4)°. Centrosymmetrically related anions form dimers by π–π stacking inter­actions, with centroid–centroid separations of 3.874 (4) Å. The crystal packing is stabilized by inter­columnar C—H⋯N hydrogen bonds.

## Related literature

For general background to the planar organic mol­ecule 7,7,8,8-tetra­cyano­quinodimethane, see: Alonso *et al.* (2005 ▶); Madalan *et al.* (2002 ▶); Liu *et al.* (2008 ▶). For the role played by the size and shape of the counter-cations in determining the ground-state electronic properties of the resulting materials, see: Ren, Meng *et al.* (2002 ▶); Ren, *et al.* (2003 ▶); Ren, Chen *et al.* (2002 ▶). For related structures, see: Liu *et al.* (2005 ▶).
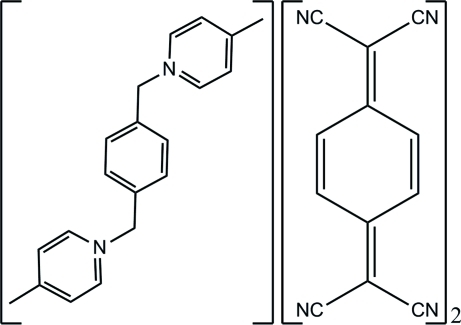


## Experimental

### 

#### Crystal data

C_20_H_22_N_2_^2+^·2C_12_H_4_N_4_^−^
*M* *_r_* = 698.78
Triclinic, 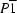

*a* = 8.5904 (12) Å
*b* = 8.6786 (11) Å
*c* = 13.3016 (17) Å
α = 101.558 (2)°
β = 106.134 (2)°
γ = 97.906 (2)°
*V* = 913.4 (2) Å^3^
*Z* = 1
Mo *K*α radiation
μ = 0.08 mm^−1^
*T* = 293 K
0.24 × 0.22 × 0.16 mm

#### Data collection

Bruker SMART APEX CCD area-detector diffractometer
Absorption correction: multi-scan (*SADABS*; Bruker, 2000 ▶) *T*_min_ = 0.981, *T*_max_ = 0.988
6854 measured reflections
3353 independent reflections
2243 reflections with *I* > 2σ(*I*)
*R*_int_ = 0.023

#### Refinement

*R*[*F* ^2^ > 2σ(*F* ^2^)] = 0.044
*wR*(*F* ^2^) = 0.126
*S* = 1.00
3353 reflections
245 parameters
H-atom parameters constrained
Δρ_max_ = 0.15 e Å^−3^
Δρ_min_ = −0.18 e Å^−3^

### 

Data collection: *SMART* (Bruker, 2000 ▶); cell refinement: *SAINT* (Bruker, 2000 ▶); data reduction: *SAINT*; program(s) used to solve structure: *SHELXS97* (Sheldrick, 2008 ▶); program(s) used to refine structure: *SHELXL97* (Sheldrick, 2008 ▶); molecular graphics: *SHELXTL* (Sheldrick, 2008 ▶); software used to prepare material for publication: *SHELXTL*.

## Supplementary Material

Crystal structure: contains datablocks I, global. DOI: 10.1107/S1600536810017113/rz2446sup1.cif

Structure factors: contains datablocks I. DOI: 10.1107/S1600536810017113/rz2446Isup2.hkl

Additional supplementary materials:  crystallographic information; 3D view; checkCIF report

## Figures and Tables

**Table 1:** Hydrogen-bond geometry (Å, °)

*D*—H⋯*A*	*D*—H	H⋯*A*	*D*⋯*A*	*D*—H⋯*A*
C16—H16⋯N3^i^	0.93	2.62	3.320 (3)	132
C17—H17⋯N2^ii^	0.93	2.47	3.204 (3)	136
C19—H19*A*⋯N3^i^	0.97	2.57	3.394 (3)	143

Symmetry codes: (i) 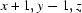; (ii) 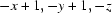.

**Fig. 1.**
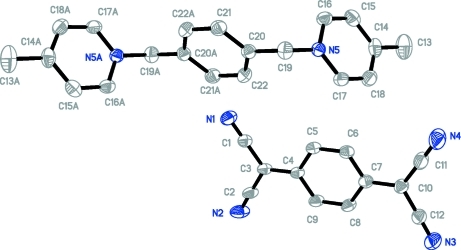
The asymmetric unit of the title compound, with the atom-numbering scheme. Displacement ellipsoids are drawn at the 30% probability level. Hydrogen atoms are omitted for clarity.

**Fig. 2.**
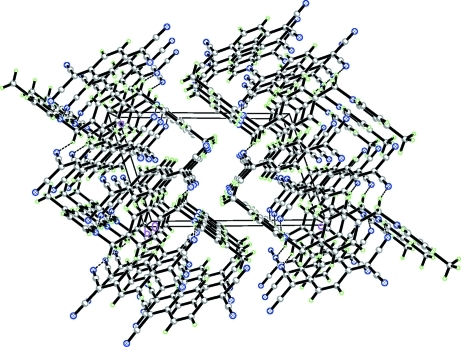
Packing diagram of the title compound viewed along the b axis. Hydrogen bonds are shown as dashed lines.

**Fig. 3.**
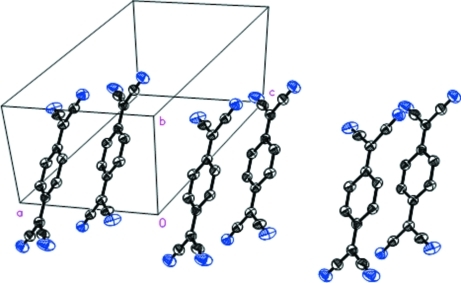
A side view of the one-dimensional anion column of the title compound. Hydrogen atoms are omitted for clarity.

## supplementary crystallographic information

### Comment

The search for new compounds with promising electronic, and magnetic properties has prompted chemists to combine different spin carriers within the same molecular or supramolecular entity (Madalan *et al.*, 2002). One of the most extensively used radicals in these studies has been the planar organic molecule 7,7,8,8-tetracyanoquinodimethane, [C_8_H_4_(CN)_4_], TCNQ, since it shows a low reduction potential which makes it a suitable acceptor in charge-transfer processes. Another significant feature of this acceptor is its tendency to overlap its π-delocalized system with those of neighbouring molecules to form stacks with different degrees of electron delocalization (Alonso *et al.*, 2005). Previous work has shown that molecular stacks of charge-transfer salts exhibit low-dimensional properties in some cases, which have intriguing anisotropic magnetic, electronic and structural characteristics (Ren, Meng *et al.*, 2002; Ren *et al.*, 2003; Liu *et al.*, 2005). Furthermore, the size and shape of the counter-cations play an important role in determining the ground-state properties of the resulting materials (Ren, Chen *et al.*, 2002; Liu *et al.*, 2008). As a result, charge-transfer salts consisting of the TCNQ anion and benzylpyridinium cations could offer the possibility of systematically studying the fundamental relationship between the stack structure and the size and steric properties of substituent groups. In this communication, the crystal structure of the title complex is reported.

The asymmetric unit of the title compound contains a half of a (C_20_H_22_N_2_)^2+^ cation and one [C_8_H_4_(CN)_4_]^-^ anion (Fig. 1). Anions and cations stack into completely segregated columns along the *b* axis, as illustrated in Fig. 2. Within an anion column, [(TCNQ)_2_]^2-^ dimers are formed by π···π stacking interactions with a centroid-to-centroid distance of 3.874 (4) Å, and adjacent units are displaced relative to each other along the direction of the shorter molecular axis of TCNQ with centroid-to-centroid separations of 6.556 (4) Å (Fig. 3). The (C_20_H_22_N_2_)^2+^ cation affords a trans conformation, with a dihedral angle between the benzene ring and the pyridine rings of 89.14 (4)°. The crystal packing is stabilized by C—H···N intercolumar linkages (Table 1).

### Experimental

1,1'-(1,4-Phenylenebis(methylene))bis(4-methylpyridin-1-ium) iodide was prepared by the direct combination of 1:2 molar equivalents of 1,1'-(1,4-phenylenebis(methylene))bis(4-methylpyridin-1-ium) chloride and NaI in a warm acetone solution at 313 K. A white precipitate was formed (NaCl), which was filtered off, and a white microcrystalline product was obtained by evaporating the filtrate. 1:2 Molar equivalents of 1,1'-(1,4-phenylenebis(methylene))bis(4-methylpyridin-1-ium) iodide and LiTCNQ were mixed directly in a methanol solution, and the mixture was refluxed for 12 h. The black microcrystalline product which formed was filtered off, washed with MeOH and dried in vacuo. Single crystals of the title compound suitable for X-ray structure analysis were obtained by diffusing diethyl ether into a MeCN solution.

### Refinement

H atoms were positioned geometrically, with C—H = 0.93, 0.97 and 0.96 Å for aromatic, methylene and methyl H atoms, respectively, and constrained to ride on their parent atoms, with Uiso(H) = xUeq(C), where x = 1.5 for methyl H and x = 1.2 for all other H atoms.

### Crystal data

**Table:**

C_20_H_22_N_2_^2+^·2C_12_H_4_N_4_^−^	*Z* = 1
*M**_r_* = 698.78	*F*(000) = 364
Triclinic, *P*1	*D*_x_ = 1.270 Mg m^−^^3^
Hall symbol: -P 1	Mo *K*α radiation, λ = 0.71073 Å
*a* = 8.5904 (12) Å	Cell parameters from 2212 reflections
*b* = 8.6786 (11) Å	θ = 2.5–26.4°
*c* = 13.3016 (17) Å	µ = 0.08 mm^−^^1^
α = 101.558 (2)°	*T* = 293 K
β = 106.134 (2)°	Block, dark green
γ = 97.906 (2)°	0.24 × 0.22 × 0.16 mm
*V* = 913.4 (2) Å^3^	

### Data collection

**Table:**

Bruker SMART APEX CCD area-detector diffractometer	3353 independent reflections
Radiation source: sealed tube	2243 reflections with *I* > 2σ(*I*)
graphite	*R*_int_ = 0.023
phi and ω scans	θ_max_ = 25.5°, θ_min_ = 1.7°
Absorption correction: multi-scan (*SADABS*; Bruker, 2000)	*h* = −10→10
*T*_min_ = 0.981, *T*_max_ = 0.988	*k* = −10→10
6854 measured reflections	*l* = −16→15

### Refinement

**Table:**

Refinement on *F*^2^	Primary atom site location: structure-invariant direct methods
Least-squares matrix: full	Secondary atom site location: difference Fourier map
*R*[*F*^2^ > 2σ(*F*^2^)] = 0.044	Hydrogen site location: inferred from neighbouring sites
*wR*(*F*^2^) = 0.126	H-atom parameters constrained
*S* = 1.00	*w* = 1/[σ^2^(*F*_o_^2^) + (0.0561*P*)^2^ + 0.1398*P*] where *P* = (*F*_o_^2^ + 2*F*_c_^2^)/3
3353 reflections	(Δ/σ)_max_ = 0.036
245 parameters	Δρ_max_ = 0.15 e Å^−^^3^
0 restraints	Δρ_min_ = −0.18 e Å^−^^3^

### Special details

**Table:**

Geometry. All esds (except the esd in the dihedral angle between two l.s. planes) are estimated using the full covariance matrix. The cell esds are taken into account individually in the estimation of esds in distances, angles and torsion angles; correlations between esds in cell parameters are only used when they are defined by crystal symmetry. An approximate (isotropic) treatment of cell esds is used for estimating esds involving l.s. planes.
Refinement. Refinement of *F*^2^ against ALL reflections. The weighted *R*-factor wR and goodness of fit *S* are based on *F*^2^, conventional *R*-factors *R* are based on *F*, with *F* set to zero for negative *F*^2^. The threshold expression of *F*^2^ > σ(*F*^2^) is used only for calculating *R*-factors(gt) etc. and is not relevant to the choice of reflections for refinement. *R*-factors based on *F*^2^ are statistically about twice as large as those based on *F*, and *R*- factors based on ALL data will be even larger.

### Fractional atomic coordinates and isotropic or equivalent isotropic displacement parameters (Å^2^)

**Table:**

	*x*	*y*	*z*	*U*_iso_*/*U*_eq_	
C1	0.3619 (2)	0.1249 (2)	−0.12031 (15)	0.0513 (5)	
C2	0.1730 (3)	0.2868 (2)	−0.18890 (17)	0.0594 (5)	
C3	0.2953 (2)	0.2639 (2)	−0.09960 (15)	0.0505 (5)	
C4	0.3415 (2)	0.3713 (2)	0.00302 (15)	0.0464 (4)	
C5	0.4625 (2)	0.3502 (2)	0.09308 (15)	0.0482 (5)	
H5	0.5125	0.2620	0.0849	0.058*	
C6	0.5072 (2)	0.4564 (2)	0.19137 (15)	0.0502 (5)	
H6	0.5873	0.4388	0.2489	0.060*	
C7	0.4357 (2)	0.5930 (2)	0.20912 (15)	0.0488 (4)	
C8	0.3129 (2)	0.6118 (2)	0.11922 (16)	0.0553 (5)	
H8	0.2618	0.6991	0.1277	0.066*	
C9	0.2674 (2)	0.5063 (2)	0.02115 (16)	0.0550 (5)	
H9	0.1854	0.5228	−0.0358	0.066*	
C10	0.4820 (2)	0.7038 (2)	0.31119 (16)	0.0547 (5)	
C11	0.5983 (3)	0.6827 (3)	0.40268 (19)	0.0699 (6)	
C12	0.4142 (2)	0.8429 (3)	0.32460 (16)	0.0595 (5)	
C13	0.7386 (3)	0.2441 (4)	0.4765 (2)	0.1005 (9)	
H13A	0.7287	0.3532	0.5007	0.151*	
H13B	0.7832	0.2031	0.5380	0.151*	
H13C	0.6314	0.1796	0.4344	0.151*	
C14	0.8517 (2)	0.2386 (3)	0.40876 (15)	0.0615 (6)	
C15	0.9129 (2)	0.1013 (3)	0.38115 (16)	0.0603 (5)	
H15	0.8838	0.0116	0.4054	0.072*	
C16	1.0148 (2)	0.0970 (2)	0.31919 (15)	0.0516 (5)	
H16	1.0558	0.0049	0.3019	0.062*	
C17	1.0016 (2)	0.3596 (2)	0.30954 (15)	0.0541 (5)	
H17	1.0331	0.4484	0.2851	0.065*	
C18	0.9013 (2)	0.3681 (3)	0.37146 (16)	0.0608 (5)	
H18	0.8649	0.4629	0.3894	0.073*	
C19	1.1638 (2)	0.2155 (2)	0.21295 (15)	0.0556 (5)	
H19A	1.2619	0.1790	0.2478	0.067*	
H19B	1.1990	0.3218	0.2043	0.067*	
C20	1.0766 (2)	0.1027 (2)	0.10308 (15)	0.0463 (4)	
C21	1.1573 (2)	−0.0062 (2)	0.05873 (16)	0.0536 (5)	
H21	1.2635	−0.0118	0.0980	0.064*	
C22	0.9181 (2)	0.1069 (2)	0.04324 (15)	0.0537 (5)	
H22	0.8614	0.1788	0.0722	0.064*	
N1	0.4125 (2)	0.0106 (2)	−0.13825 (15)	0.0691 (5)	
N2	0.0727 (3)	0.3070 (2)	−0.25956 (17)	0.0860 (7)	
N3	0.3576 (3)	0.9543 (2)	0.33418 (17)	0.0811 (6)	
N4	0.6931 (3)	0.6641 (3)	0.47670 (18)	0.1053 (8)	
N5	1.05663 (17)	0.22537 (17)	0.28280 (11)	0.0450 (4)	

### Atomic displacement parameters (Å^2^)

**Table:**

	*U*^11^	*U*^22^	*U*^33^	*U*^12^	*U*^13^	*U*^23^
C1	0.0510 (10)	0.0456 (11)	0.0541 (12)	0.0084 (9)	0.0109 (9)	0.0147 (9)
C2	0.0663 (13)	0.0334 (10)	0.0669 (14)	0.0076 (9)	0.0044 (11)	0.0116 (10)
C3	0.0517 (10)	0.0386 (10)	0.0582 (12)	0.0100 (8)	0.0089 (9)	0.0170 (9)
C4	0.0477 (10)	0.0360 (9)	0.0550 (11)	0.0079 (8)	0.0131 (9)	0.0154 (9)
C5	0.0449 (10)	0.0412 (10)	0.0595 (12)	0.0126 (8)	0.0129 (9)	0.0171 (9)
C6	0.0451 (10)	0.0483 (11)	0.0552 (12)	0.0115 (8)	0.0091 (9)	0.0170 (9)
C7	0.0455 (10)	0.0453 (10)	0.0563 (12)	0.0085 (8)	0.0160 (9)	0.0149 (9)
C8	0.0569 (11)	0.0444 (11)	0.0645 (13)	0.0195 (9)	0.0148 (10)	0.0133 (10)
C9	0.0552 (11)	0.0450 (11)	0.0607 (13)	0.0171 (9)	0.0060 (10)	0.0170 (10)
C10	0.0542 (11)	0.0528 (12)	0.0578 (12)	0.0164 (9)	0.0174 (10)	0.0119 (10)
C11	0.0687 (14)	0.0693 (15)	0.0610 (15)	0.0259 (11)	0.0103 (12)	−0.0010 (11)
C12	0.0608 (12)	0.0614 (14)	0.0584 (13)	0.0173 (11)	0.0207 (10)	0.0136 (11)
C13	0.0858 (17)	0.130 (2)	0.0825 (18)	0.0091 (16)	0.0458 (15)	0.0010 (16)
C14	0.0526 (11)	0.0783 (15)	0.0427 (11)	0.0085 (10)	0.0121 (9)	−0.0015 (10)
C15	0.0645 (12)	0.0624 (13)	0.0504 (12)	0.0020 (10)	0.0153 (10)	0.0179 (10)
C16	0.0573 (11)	0.0398 (10)	0.0558 (12)	0.0097 (8)	0.0135 (9)	0.0137 (9)
C17	0.0651 (12)	0.0447 (11)	0.0493 (11)	0.0178 (9)	0.0093 (10)	0.0130 (9)
C18	0.0618 (12)	0.0584 (13)	0.0556 (13)	0.0218 (10)	0.0106 (10)	0.0042 (10)
C19	0.0538 (11)	0.0557 (12)	0.0555 (12)	0.0047 (9)	0.0199 (10)	0.0105 (10)
C20	0.0470 (10)	0.0473 (10)	0.0496 (11)	0.0104 (8)	0.0197 (9)	0.0165 (8)
C21	0.0462 (10)	0.0610 (12)	0.0566 (12)	0.0184 (9)	0.0149 (9)	0.0180 (10)
C22	0.0551 (11)	0.0543 (12)	0.0576 (12)	0.0216 (9)	0.0234 (10)	0.0120 (10)
N1	0.0723 (11)	0.0566 (11)	0.0773 (13)	0.0242 (9)	0.0182 (10)	0.0145 (9)
N2	0.0910 (14)	0.0543 (11)	0.0851 (14)	0.0136 (10)	−0.0172 (12)	0.0205 (10)
N3	0.0905 (14)	0.0739 (13)	0.0845 (14)	0.0362 (11)	0.0307 (12)	0.0143 (11)
N4	0.1095 (17)	0.1122 (18)	0.0688 (14)	0.0548 (15)	−0.0072 (13)	−0.0060 (13)
N5	0.0505 (8)	0.0400 (8)	0.0415 (8)	0.0092 (7)	0.0110 (7)	0.0083 (7)

### Geometric parameters (Å, °)

**Table:**

C1—N1	1.145 (2)	C13—H13B	0.9600
C1—C3	1.415 (3)	C13—H13C	0.9600
C2—N2	1.146 (2)	C14—C18	1.379 (3)
C2—C3	1.419 (3)	C14—C15	1.391 (3)
C3—C4	1.406 (3)	C15—C16	1.358 (3)
C4—C5	1.413 (2)	C15—H15	0.9300
C4—C9	1.419 (2)	C16—N5	1.344 (2)
C5—C6	1.359 (2)	C16—H16	0.9300
C5—H5	0.9300	C17—N5	1.337 (2)
C6—C7	1.416 (2)	C17—C18	1.347 (3)
C6—H6	0.9300	C17—H17	0.9300
C7—C8	1.412 (3)	C18—H18	0.9300
C7—C10	1.413 (3)	C19—N5	1.477 (2)
C8—C9	1.353 (3)	C19—C20	1.507 (3)
C8—H8	0.9300	C19—H19A	0.9700
C9—H9	0.9300	C19—H19B	0.9700
C10—C11	1.406 (3)	C20—C22	1.383 (2)
C10—C12	1.412 (3)	C20—C21	1.380 (2)
C11—N4	1.146 (3)	C21—C22^i^	1.380 (3)
C12—N3	1.141 (2)	C21—H21	0.9300
C13—C14	1.497 (3)	C22—C21^i^	1.380 (3)
C13—H13A	0.9600	C22—H22	0.9300
			
N1—C1—C3	178.6 (2)	C18—C14—C15	116.78 (18)
N2—C2—C3	178.5 (2)	C18—C14—C13	122.2 (2)
C4—C3—C2	121.18 (16)	C15—C14—C13	121.1 (2)
C4—C3—C1	123.07 (16)	C16—C15—C14	120.68 (19)
C2—C3—C1	115.72 (17)	C16—C15—H15	119.7
C3—C4—C5	122.03 (16)	C14—C15—H15	119.7
C3—C4—C9	121.27 (16)	N5—C16—C15	120.27 (18)
C5—C4—C9	116.70 (17)	N5—C16—H16	119.9
C6—C5—C4	121.22 (16)	C15—C16—H16	119.9
C6—C5—H5	119.4	N5—C17—C18	120.89 (19)
C4—C5—H5	119.4	N5—C17—H17	119.6
C5—C6—C7	122.16 (17)	C18—C17—H17	119.6
C5—C6—H6	118.9	C17—C18—C14	121.10 (19)
C7—C6—H6	118.9	C17—C18—H18	119.4
C8—C7—C10	121.36 (17)	C14—C18—H18	119.4
C8—C7—C6	116.30 (17)	N5—C19—C20	112.18 (14)
C10—C7—C6	122.33 (17)	N5—C19—H19A	109.2
C9—C8—C7	121.90 (17)	C20—C19—H19A	109.2
C9—C8—H8	119.1	N5—C19—H19B	109.2
C7—C8—H8	119.1	C20—C19—H19B	109.2
C8—C9—C4	121.70 (17)	H19A—C19—H19B	107.9
C8—C9—H9	119.1	C22—C20—C21	118.33 (17)
C4—C9—H9	119.1	C22—C20—C19	121.95 (17)
C11—C10—C12	117.16 (18)	C21—C20—C19	119.70 (16)
C11—C10—C7	122.08 (17)	C20—C21—C22^i^	120.67 (17)
C12—C10—C7	120.74 (18)	C20—C21—H21	119.7
N4—C11—C10	179.3 (2)	C22^i^—C21—H21	119.7
N3—C12—C10	179.1 (2)	C20—C22—C21^i^	121.00 (17)
C14—C13—H13A	109.5	C20—C22—H22	119.5
C14—C13—H13B	109.5	C21^i^—C22—H22	119.5
H13A—C13—H13B	109.5	C17—N5—C16	120.25 (16)
C14—C13—H13C	109.5	C17—N5—C19	120.70 (15)
H13A—C13—H13C	109.5	C16—N5—C19	119.05 (15)
H13B—C13—H13C	109.5		
			
C2—C3—C4—C5	179.72 (17)	C18—C14—C15—C16	−0.8 (3)
C1—C3—C4—C5	1.8 (3)	C13—C14—C15—C16	179.7 (2)
C2—C3—C4—C9	0.1 (3)	C14—C15—C16—N5	−0.6 (3)
C1—C3—C4—C9	−177.77 (18)	N5—C17—C18—C14	−0.3 (3)
C3—C4—C5—C6	179.13 (17)	C15—C14—C18—C17	1.3 (3)
C9—C4—C5—C6	−1.2 (2)	C13—C14—C18—C17	−179.2 (2)
C4—C5—C6—C7	0.0 (3)	N5—C19—C20—C22	46.7 (2)
C5—C6—C7—C8	1.0 (3)	N5—C19—C20—C21	−134.85 (17)
C5—C6—C7—C10	−179.94 (18)	C22—C20—C21—C22^i^	0.7 (3)
C10—C7—C8—C9	−179.89 (19)	C19—C20—C21—C22^i^	−177.80 (17)
C6—C7—C8—C9	−0.8 (3)	C21—C20—C22—C21^i^	−0.7 (3)
C7—C8—C9—C4	−0.4 (3)	C19—C20—C22—C21^i^	177.76 (17)
C3—C4—C9—C8	−178.94 (18)	C18—C17—N5—C16	−1.2 (3)
C5—C4—C9—C8	1.4 (3)	C18—C17—N5—C19	178.69 (17)
C8—C7—C10—C11	176.93 (19)	C15—C16—N5—C17	1.7 (3)
C6—C7—C10—C11	−2.1 (3)	C15—C16—N5—C19	−178.25 (16)
C8—C7—C10—C12	−4.2 (3)	C20—C19—N5—C17	−110.79 (18)
C6—C7—C10—C12	176.78 (18)	C20—C19—N5—C16	69.1 (2)

Symmetry codes: (i) −*x*+2, −*y*, −*z*.

### Hydrogen-bond geometry (Å, °)

**Table:**

*D*—H···*A*	*D*—H	H···*A*	*D*···*A*	*D*—H···*A*
C16—H16···N3^ii^	0.93	2.62	3.320 (3)	132
C17—H17···N2^iii^	0.93	2.47	3.204 (3)	136
C19—H19A···N3^ii^	0.97	2.57	3.394 (3)	143

Symmetry codes: (ii) *x*+1, *y*−1, *z*; (iii) −*x*+1, −*y*+1, −*z*.
